# On the function of biosynthesized cellulose as barrier against bacterial colonization of VAD drivelines

**DOI:** 10.1038/s41598-021-98220-4

**Published:** 2021-09-21

**Authors:** Julius Kaemmel, Aldo Ferrari, Francesco Robotti, Simone Bottan, Fritz Eichenseher, Tanja Schmidt, Mercedes Gonzalez Moreno, Andrej Trampuz, Jaime-Jürgen Eulert-Grehn, Christoph Knosalla, Evgenij Potapov, Volkmar Falk, Christoph Starck

**Affiliations:** 1 Department of Cardiothoracic and Vascular Surgery, German Heart Center Berlin, Augustenburger Platz 1, 13353 Berlin, Germany; 2 Hylomorph AG, Technoparkstrasse 1, 8005 Zurich, Switzerland; 3 Wyss Zurich, Zurich, Switzerland; 4 Food Microbiology Laboratory, ETH Zurich, Schmelzbergstrasse 7, 8092 Zurich, Switzerland; 5 Forschungseinrichtungen für Experimentelle Medizin, Charité-Universitätsmedizin Berlin, Berlin, Germany; 6 Charité-Universitätsmedizin Berlin, corporate Member of Freie Universität Berlin and Humboldt-Universität zu Berlin, Center for Musculoskeletal Surgery, Augustenburger Platz 1, 13353 Berlin, Germany; 7 Berlin Institute of Health at Charité-Universitätsmedizin Berlin, BIH Center for Regenerative Therapies (BCRT), Charitéplatz 1, 10117 Berlin, Germany; 8 DZHK (German Centre for Cardiovascular Research), Partner Site Berlin, Berlin, Germany; 9 Charité—Universitätsmedizin Berlin, corporate Member of Freie Universität Berlin, Humboldt-Universität zu Berlin and Berlin Institute of Health, Berlin, Germany; 10 Department of Cardiovascular Surgery, Charité-Universitätsmedizin Berlin, corporate Member of Freie Universität Berlin and Humboldt-Universität zu Berlin, Berlin, Germany

**Keywords:** Biomaterials, Cardiac device therapy

## Abstract

Bacterial colonization of drivelines represents a major adverse event in the implantation of left ventricular assist devices (L-VADs) for the treatment of congestive heart failure. From the external driveline interface and through the skin breach, pathogens can ascend to the pump pocket, endangering the device function and the patient’s life. Surface Micro-Engineered Biosynthesized cellulose (BC) is an implantable biomaterial, which minimizes fibrotic tissue deposition and promotes healthy tissue regeneration. The topographic arrangement of cellulose fibers and the typical material porosity support its potential protective function against bacterial permeation; however, this application has not been tested in clinically relevant animal models. Here, a goat model was adopted to evaluate the barrier function of BC membranes. The external silicone mantle of commercial L-VAD drivelines was implanted percutaneously with an intervening layer of BC to separate them from the surrounding soft tissue. End-point evaluation at 6 and 12 weeks of two separate animal groups revealed the local bacterial colonization at the different interfaces in comparison with unprotected driveline mantle controls. The results demonstrate that the BC membranes established an effective barrier against the bacterial colonization of the outer driveline interface. The containment of pathogen infiltration, in combination with the known anti-fibrotic effect of BC, may promote a more efficient immune clearance upon driveline implantation and support the efficacy of local antibiotic treatments, therefore mitigating the risk connected to their percutaneous deployment.

## Introduction

Congestive heart failure is a common and severe medical condition, with 2.2% prevalence in the United States^1^. Individuals in the age of 40 have a lifetime risk of 20% for developing heart failure^2^. In the frame of an ageing Western population, these figures are bound to further increase^3^.

Due to the shortage of donor organs and the limited therapeutic options for patients with end-stage heart failure, left ventricular assist devices (L-VADs) have emerged as a viable alternative to heart transplantation^4^. Nowadays, VADs are implanted with different therapeutic goals, ranging from bridge-to-transplantation to destination therapy^5^. Patients treated with continuous flow L-VADs have 1-year survival rates comparable to those patients receiving an orthotopic heart transplant^6,7^. However, these figures diverge at 3 years, with patients under mechanical support showing significantly reduced survival^6,7^. After 1 year on mechanical circulatory support approximately 19% of patients develop at least one driveline infection^8^. In general, VAD infections are associated with reduced survival of patients^8,9^.

State of the art L-VADs [e.g. HeartMate3 (HM3; Abbott, Chicago, IL, USA) and HeartWare HVAD (HW; Medtronic, Minneapolis, MN, USA)] rely on an external power source and control unit thus requiring a percutaneous driveline. The associated exit sites represent a breach in the skin barrier function against pathogens. The superficial colonization of the driveline, able to ascend and reach the pump pocket, is a common adverse event connected to VADs for mechanical circulatory support^10^. According to the International Society for Heart and Lung Transplantation (ISHLT), VAD infections are classified as follows: VAD-specific infections, VAD-related infections and non-VAD infections. Infections involving the driveline and the adjacent tissue are accounted as VAD-specific infections^11^.

These infections are mostly caused by Gram-positive or Gram-negative bacteria such as *Staphylococcus aureus, Staphylococcus epidermidis and Pseudomonas aeruginosa*^12^, whereas fungal infections only account for a small fraction of them.

In addition to this, and in all cases even if to a variable extent, the interaction between an implanted medical device and the body tissues elicits an acute and chronic inflammation, the foreign body reaction (FBR). Over time, FBR leads to the encapsulation of the synthetic materials in a dense fibrous tissue and establishes an environment with poor vascularization and reduced immune surveillance^13–15^.

This further complicates the picture, as the silicone interface of the drivelines becomes rapidly encapsulated. In this scenario, the interplay between FBR and implant infection is twofold. First, the dysregulated immune response to foreign bodies, leading to fibrosis, hampers the local tissue ability to react to contaminating pathogens and is conducive to the establishment of a persisting infection around the implant^14,15^. The colonization of soft tissues in the surgical pocket can additionally generate niches for pathogen survival. These have been shown to cause relapsing infections upon revision of contaminated implants^14^.

Second, the metallic or silicone surfaces of most implants are prone to bacterial adhesion and biofilm formation^15^. The process is supported by the aspecific protein fouling of abiotic surfaces which mediates bacteria docking via adhesins, binding to matrix proteins such as collagen, fibronectin, and fibrinogen. The fate of the implant is then decided by whether host cells can integrate the biomaterial before bacteria colonize the surface. If bacteria win this ‘race to the surface’^15^ a biofilm is established, which cannot be cleared by the host immune system and is often resistant to antimicrobial treatments. Persistent biofilms cause chronic inflammations in the surgical pocket and represent a root cause of implant failure and death. Altogether, driveline infections and their fibrotic encapsulation remain major challenges tainting the success of VADs as viable treatment for heart failure. Minimizing FBR and limiting biofilm formation shall be therefore regarded as key measure against recurrent implant infections.

Biosynthesized cellulose (BC) is a naturally occurring polymer that has received increasing consideration as promising implantable biomaterial^16^. Bacterial fermentation of sugars at the interface with air generates free-form layers of a cellulose hydrogel containing > 95% of water. The process can be harnessed by up-scalable fabrication protocols, which control the distribution of cellulose nanofibers, the resulting nanoscale porosity, and the microscale surface geometry^17^. Currently, BC is approved as *dura mater* substitute^18^, and chronic in vivo tests demonstrate its efficacy in minimizing the foreign body reaction against cardiac implantable electronic devices^13^. Surface Micro-Engineered BC^17^ has shown excellent antifouling and antiadhesive properties significantly reducing fibrotic tissue deposition around soft tissue implants^13^. In addition, it establishes a porous interface improving the efficacy of antibiotic molecules, as compared to metals or silicones^19^. However, the role of BC as barrier against bacterial infiltration and colonization remains to be proven both in vitro and in vivo.

Here, we tested the protective effect of BC layers to prevent bacterial colonization of the L-VAD driveline external silicone interface (the silicone driveline mantle; s.d.m.) in a large animal model in vivo. The study rationale leverages on the antifibrotic effect of the protective material coupled with its intrinsic nano-scale architecture blocking bacterial infiltration and supporting immune clearance. To this end, we percutaneously implanted the silicone mantle of L-VAD drivelines with and without BC protection and evaluated the dynamics of bacterial infiltration at their external interface. Specifically, two time points at 6- and 12-weeks post-implantation were analyzed, and bacterial colonization was detected by microbiological screening.

## Materials and methods

### Biosynthesized cellulose fabrication and characterization

The Surface Micro-Engineered BC used in this study was synthesized by a strain of the bacterium *Acetobacter xylinum* in static culture. The wild type Acetobacter xylinum strain ATCC-700178 (LGC Standards, Wesel, Germany) was used for BC fermentation^17^. The bacteria were grown in a medium prepared as reported in Table 1 and sterilized by autoclaving. The resulting BC membranes were made of a three-dimensional network of randomly arranged cellulose nanoribbons, forming a multi-layered hydrogel with high porosity^17^. The average pore diameter was < 500 nm.

**Table 1:** Composition of culture medium for *Acetobacter xylinum*.

Additive	Molecular formula	Quantity
d-Glucose	C_6_H_12_O_6_	20 g
Diazanium sulfate (ammonium sulfate)	(NH_4_)2SO_4_	3.5 g
Potassium dihydrogen phosphate (phosphoric acid)	KH_2_PO_4_	7 g
Sodium phosphate dibasic dodecahydrate	(Na_2_HPO_4_)12H_2_O	3.4 g
Magnesium sulfate heptahydrate	(MgSO_4_)7H_2_O	2.1 g
Boric acid	H_3_BO_3_	4.3 mg
Ferrous sulfate heptahydrate	(FeSO_4_)7H_2_O	9.5 mg
Pyridine-3-carboxamide (nicotinamide)	C_6_H_6_N_2_O	0.7 mg
Ethanol	C_2_H_5_OH	6 ml

Additives for 1 l of medium in Milli-Q filtered water.

The BC membranes were harvested after 1 week of incubation at 27 °C. BC purification was performed as reported in Bottan et al.^17^. Briefly, at the end of the culturing period a thick (3–4 mm) cellulose layer was formed. BC substrates were then harvested. To remove bacteria from the BC the pellicles were washed in NaOH 1 M for 80 min at 80 °C, and subsequently in deionized (DI) water at room temperature (RT) until neutral pH was reestablished. The BC substrates were fully hydrated upon harvesting from the bacterial fermentation culture. This stage is defined as nondehydrated bacterial cellulose (NDH). The NDH substrates were then washed and dehydrated overnight at RT. Dehydrated cellulose substrates (DH) were then rehydrated with DI water. Finally, the rehydrated substrates (RH) were autoclaved (121 °C, 1.1 bar for 15 min) and stored in PBS at 4 °C. Membranes had a residual endotoxin content < 20 EU/device, as prescribed for fully implantable medical devices^13^. In particular, sterile biosynthetic cellulose sheets measuring 10 × 20 cm were used for the animal experiments.

The surface porosity (Φ) was measured on planar images at high magnification (25,000×) as the ratio of the area of pores divided by the area of the field of view (Supplementary Fig. 1). The area of the pores was calculated using the Analyze Particle tool of ImageJ on a binary mask obtained from the SEM image setting a signal intensity threshold. To obtain a value for the average pore diameter, Φ was divided by the number of individual pores detected from the processed image. This evaluation was performed on multiple fields of view to obtain a standard deviation for the average pore diameter.

After preparation, the BC substrates were characterized using scanning electron microscope (SEM) imaging. To prepare the samples for SEM, the substrates were washed twice in Milli-Q water, and then rinsed for 10 min in increasing concentrations of filtered ethanol (30, 50, 70, 90 and 95%). They were then rinsed twice in 100% ethanol for 15 min each. Ethanol dehydration was followed by gradual replacement with hexamethyldisilazane (Sigma-Aldrich) that was let to evaporate in a fume hood overnight. Samples were finally coated with a 5 nm-thick film of gold/palladium (60/40 wt%). The substrates were imaged using a JEOL JSM-7500FA scanning electron microscope (SEM), equipped with a cold field emission gun (FEG). Initially, low magnification images at 30° tilt angle were acquired, to allow for perspective 3D imaging. The BC nanofibers were imaged at higher magnification and resolution, operating the machine at an acceleration voltage 2 kV in order to minimize any possible beam damage effect. In both cases the SEM imaging was performed by collecting secondary electron (SE) signal.

### In vitro* permeation assay*

A custom-developed setup was exploited for the permeation assay (Fig. 3). A test membrane (i.e. the BC membrane) was mounted to separate the two chambers (Fig. 3A). The upper chamber was then filled with a stabilized suspension of 2 µm microparticles (SPHERO Fluorescent Light Yellow Particles 1%w/v 1.97 µm, Spherotech Inc.), while the lower chamber was filled with an equivalent volume of pure solvent. After 24 h incubation at room temperature, the test membrane was retrieved and the number of beads permeating from the upper to the lower chamber was evaluated at different time points by inspecting the membrane surface by means of bright-field microscopy.

For bacterial colony formation, cubes of agar with lateral size of 1 cm were prepared and inoculated with a solution of *S. aureus* (Fig. 3C). The agar cubes were then leaned on a BC membrane separating them from an agar plate. Samples were incubated overnight at 37 °C and the colonization of the agar plate surface (i.e. by bacteria crossing the BC membrane) was evaluated. BC membranes in which an array of holes was created by puncturing with a syringe tip were included as internal controls, following the same experimental scheme.

### Experimental animals

The animal study was performed on adult non-gravid, horned and dehorned female goats (German improved white goat/Preclinics GmbH) on the basis of the governmental permit G 0099/18 issued by LaGeSo (Berlin, Germany) and according to the guidelines of the EU directive 2010/63, the German animal protection act (TierSchG) and the regulation for the protection of laboratory animals (TierSchVersV). The animal study complies with the ARRIVE guidelines.

**Figure 1:**
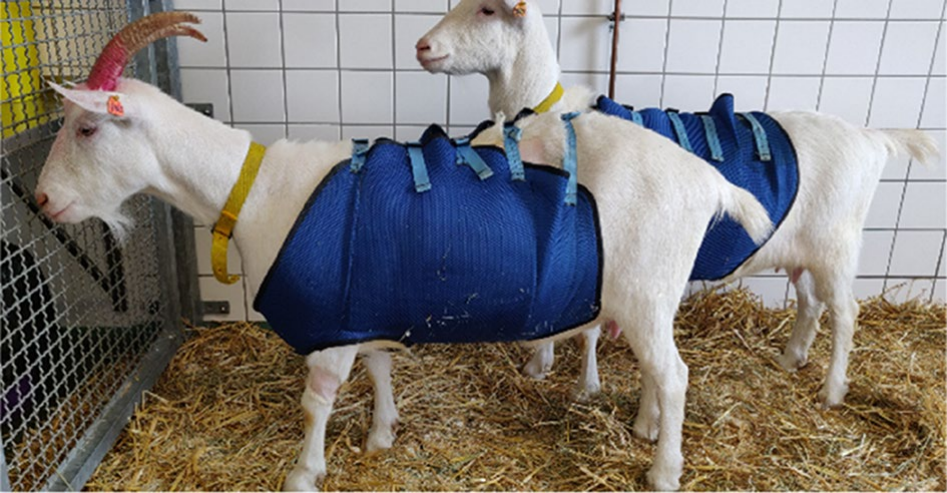
Experimental animal wearing a protective vest to secure the integrity of wound dressings.

Large animals were necessary for this study since the implanted s.d.m. (i.e. the external silicon layer of commercial L-VAD drivelines) measured 6–10 cm in length. Goats represent the most adequate model. They can be easily trained and handled. Using protective vests, self-mutilation of the implants can be prevented even in group housing. For this study, the protective vests for maintenance of proper wound hygiene were fabricated based on the design published by Grosshauser et al.^20^ (Fig. 1).

A total of eight (n = 8) animals were included in the study. Animals were divided into two groups, a 6-week (animals Nr. 1–4) and a 12-week (animals Nr. 5–8) endpoint group based on the time span between the implantation and explantation of the s.d.m. Each animal (6-week and 12-week group) received four s.d.m. (n = 4) to reduce the total number of animals. On both flanks of the animal, one control s.d.m. without BC coating and one BC covered s.d.m. were implanted following a staggered scheme. In total, each group of animals received sixteen individual s.d.m. (n = 16), i.e. eight control s.d.m. (n = 8) and eight BC covered s.d.m. (n = 8). No animal was excluded during this study.

### Housing, husbandry and animal welfare

Animals were allowed to adapt to the new environment, personnel and the protective vests for 2 weeks before the surgery. In addition, all animals were trained to walk the path from the stable to the room where the anesthesia induction was performed and were acclimated to the inhalation masks. Training as well as later induction of anesthesia was performed in groups of two animals. This approach was chosen to reduce stress to individual animals. In particular, induction of anesthesia directly via Isofluran application using an inhalation mask rendered premedication by intramuscular injection unnecessary.

In general, animals were accommodated in stables in groups of 2–4. Each stable was equipped with an automated water dispenser and appropriate enrichment. Animals were fed with hay and feed pellets. Upon study-associated manipulations or health checkups, animals were additionally fed with carrots and apples. The bedding consisted of a combination of sawdust and straw. Pens were cleaned and disinfected once a week. Temperature was kept at 18 ± 2 °C and air humidity at 55 ± 10%. Stables were illuminated by daylight, ensuring a natural circadian rhythm.

All animals were inspected daily by veterinarians and animal care attendants. Each animal was weighted weekly. Post implantation, wounds were inspected regularly at intervals ranging from daily to once a week. The frequency of wound controls and the associated dressing changes were primarily dependent on the condition of the wound dressings. General health and wound condition were documented at every inspection.

### Perioperative management for implantation

All animals were fasted for 12 h before anesthesia induction. Anesthesia was induced by inhalation of Isoflurane (3.0–5.0%) using a facemask connected to the respirator. Afterwards, an IV access was established on an ear vein and a distal leg vein. Sedation was intensified by an IV bolus (10–15 mg/kg) of Sodium Thiopental. Following endotracheal intubation, anesthesia was maintained with inhaled Isofluran (0.8–1.5%) and IV Fentanyl (1–5 µg/kg bolus/1–5 µg/kg/h). All animals received a Fentanyl (75 µg/h) skin patch to one foreleg before surgery. Moreover, an oropharyngeal temperature probe and a ruminal tube were placed. All animals received preoperatively a single shot of Ampicillin/Sulbactam (2000 mg/1000 mg) intravenously. Continued antibiotic prophylaxis until healing of the wound was ensured by an intramuscular injection of Amoxicillin (15 mg/kg) before the end of surgery. The injection with Amoxicillin was repeated at 48 and 96 h after surgery. During anesthesia heart rate and respiratory rate were monitored continuously. Proper mechanical ventilation was ensured by capnometry.

### Implantation procedure

Commercial s.d.m. of HeartMate3 (HM3; Abbott, Chicago, IL, USA) drivelines were used in this study. The external silicon layer of the drivelines (i.e. the s.d.m.) was divided in 6–10 cm long pieces. Previous studies indicated an increase in the risk of infection by a skin-velour interface compared to a skin-silicone interface, hence the velour portion of the s.d.m. was discarded in this study^21,22^. Sterile BC sheets were unfolded on wet sterile gauze and aligned in a manner that the rounded edge was situated at the right top corner. This approach ensures correct application of the cellulose to the s.d.m.

**Figure 2:**
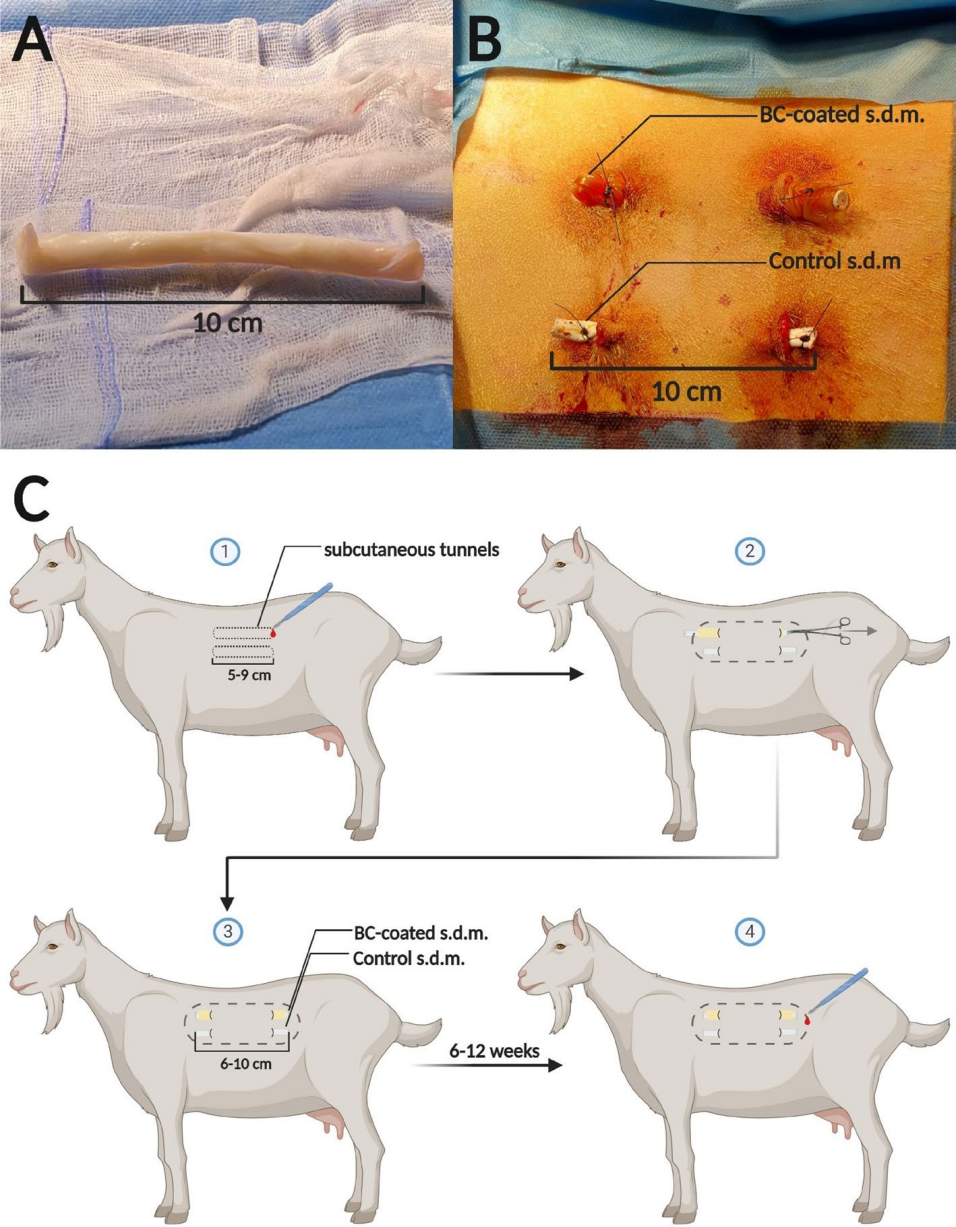
Application of biosynthesized cellulose (BC) to the silicone mantle (s.d.m) of L-VAD-drivelines and implantation process. (**A**) Original s.d.m. wrapped in BC. Both ends of overlapping BC were trimmed to the length of the s.d.m. (**B**) Surgical site at the end of the procedure. S.d.m. are secured at the exit sites by a single purse string suture. (**C**) Preparation of subcutaneous tunnels and insertion of s.d.m. Arrangement of implants at the animals flank. Dotted line represents future incision for the explantation of the s.d.m.

The BC membranes adopted for this study exhibited excellent tensile strength and proved very conformable^13^. These properties allow the material to wrap around the high curvature of the silicone drivelines used for this study. The application of the BC protective layer was performed by tightly rolling the membrane around the driveline silicone surface. Due to the properties of hydrated BC, the membranes were tightly adhering to the s.d.m. after application. The process was performed without damaging or tearing the BC layer. Afterwards, the protruding ends of the cellulose were trimmed to the exact length of the s.d.m. on both sides (Fig. 2A). After implantation of the BC-protected s.d.m. into the subcutaneous tunnel, the surrounding tissue naturally applied sufficient pressure to the implant to maintain the BC membrane in place without unfolding. A single suture at the end of each driveline tied the membrane to the silicone driveline outer layer and further stabilized the implant configuration. The correct configuration was retrieved upon explantation of the test articles for the ensuing analysis.

Implantation was performed for all animals in aseptic technique. Prior to surgery, both flanks of the animals were shaved using hair clippers. Thereafter the skin of both flanks up to the spine was thoroughly disinfected using a 7.5% povidone-iodine-solution. Surgical drapes were used to create a rectangular surgical field measuring approximately 15 × 20 cm on both flanks of the animal.

Two subcutaneous tunnels were created on each side of the animal by incising the skin at four points with a scalpel and subsequent atraumatic preparation in the subcutaneous tissue with surgical scissors. Each tunnel measured 5–9 cm in length. The two tunnels on the same animal side were approximately 4–6 cm apart. Implantation of control s.d.m. and BC covered counterparts was performed by channeling through the subcutaneous tunnels and securing them with a non-absorbable 2–0 Ethilon (Ethicon, USA) purse-string suture at each exit site (Fig. 2B, C). For additional stability, the s.d.m. ends protruding from the subcutaneous tissue were tied to the sutures securing the exit sites (Fig. 2B).

### Dressing changes

Dressing changes were performed aseptically either by using a touchless technique or by wearing sterile surgical gloves. Adhesions and contaminants were removed using sterile 0.9% saline solution and sterile compresses. Wounds were covered using sterile compresses and adhesive bandage. Edges of the bandage were further secured using an extra bandage layer. The first wound control and dressing change was performed within the first three postoperative days. Consecutive dressing changes were performed regularly every 1–7 days depending on the integrity of the wound closure material.

### Sample collection

Animals were euthanized directly after being under deep anesthesia induced by inhalation of Isoflurane (5.5%) followed by IV administration of 2500 mg Sodium Thiopental, 0.5 mg Fentanyl, 2 mg Pancuronium Bromide and 60 ml of 7.45% potassium chloride solution. The s.d.m. and the adjacent tissue specimens were then gathered directly.

An approximately 3 cm thick (dissected to the muscle fascia) square tissue section (10 × 10 cm) was cut out of the skin utilizing a scalpel. Importantly, the tissue size was selected such that the edges of the sample were at least 2 cm away from the implant at all points. Once explanted, the two tissue specimens (from both flanks of the animal, containing all 4 s.d.m.), were transferred to another sterile instrument table for further dissection and sample collection. Specifically, each sample (s.d.m. and surrounding tissue) was dissected longitudinally into three sections. Two sections contained the exit sites (left and right, where the s.d.m. exits the skin) and the middle section containing the subcutaneous part of the implant. The middle section measured approximately 1 cm in length.

The middle section was cut longitudinally in order to separate the s.d.m. from the adjacent tissue. Control s.d.m. were freed from the tissue and transferred into 15 ml Falcon tubes filled with 3 ml sterile 0.9% NaCl solution. BC-covered s.d.m. were separated prior to transfer into 15 ml Falcon tubes filled with 3 ml sterile 0.9% NaCl solution. Therefore, the BC was dissected and peeled off the corresponding s.d.m. This separation resulted in three distinct groups for microbiologic testing: (1) control bare s.d.m. (B-s.d.m.), (2) BC-coated s.d.m. deprived of BC (BCB-s.d.m.) and (3) BC. Transfer of implant specimens into Falcon tubes was performed under sterile conditions.

### Microbiology

Wound swabs collected from the subcutaneous tunnel and corresponding exit sites prior to the s.d.m. implantation were sent to a specialized veterinarian laboratory (LABOKLIN, Germany) for microbiologic testing.

Post-collection specimens were processed in a microbiology laboratory within 6 h. Falcon tubes containing the implants immersed in 0.9% NaCl were vortexed for 30 s (Vortex Genie 2, Scientific Industries, Bohemia NY, USA), followed by sonication in an ultrasound bath at 40 kHz and 0.2 W/cm^2^ (BactoSonic/BANDELIN electronic GmbH & Co. KG) for 1 min and another 30 s vortexing. Finally, 100 µl of the sonication fluid were plated on a brain heart infusion (BHI; BD, Le Pont de Claix, France) agar and incubated for 24 h at 37 °C. Sterile not implanted BC (same batch as the implanted BC) and a sterile driveline mantle of a HeartWare HVAD (HW; Medtronic, Minneapolis, MN, USA) driveline were used as negative controls.

### Software

The cartoons in Fig. 2 were created with Biorender.com.

## Results

### In vitro barrier function of BC

**Figure 3:**
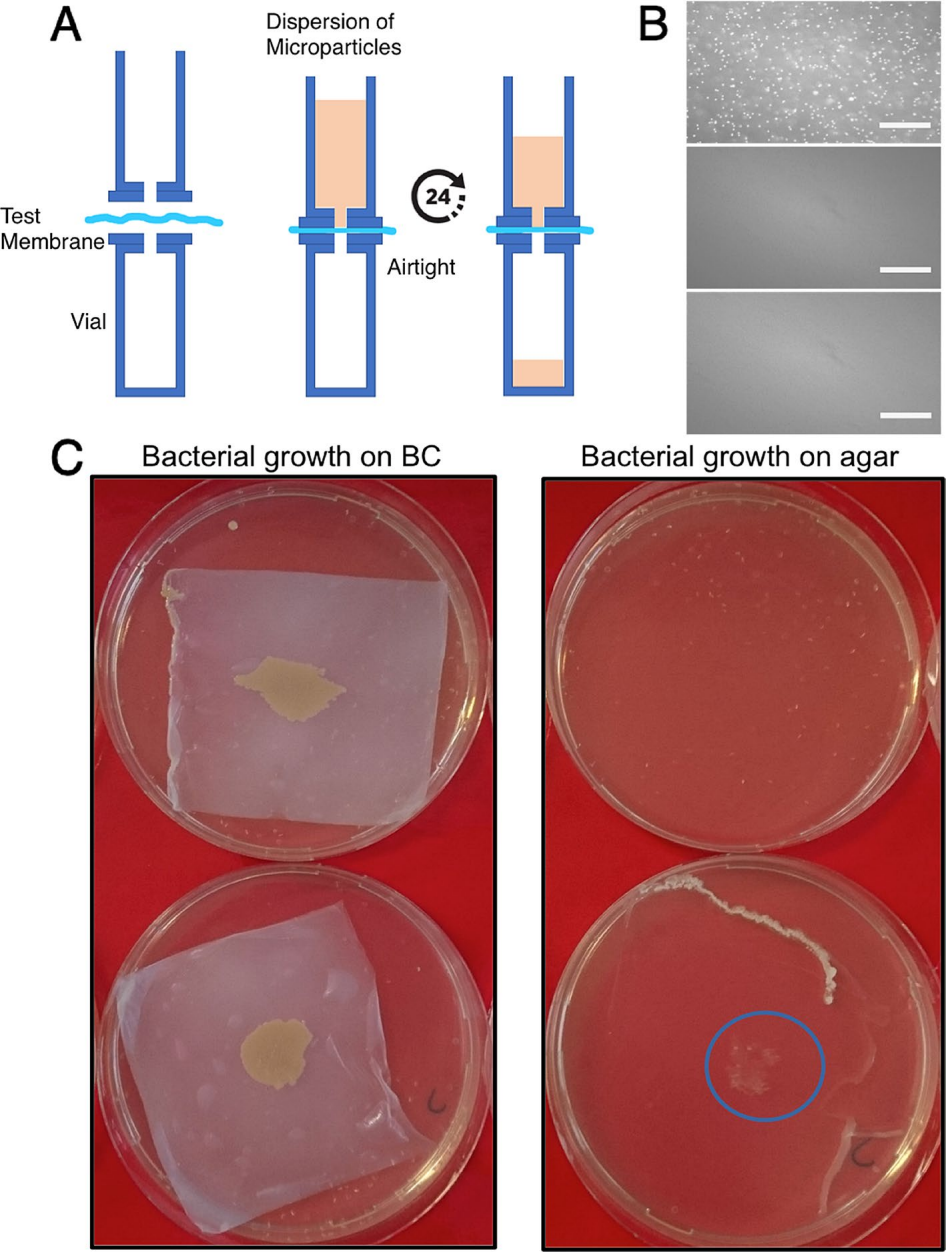
In vitro test of BC barrier function. (**A**) Experimental settings. A test BC membrane separates two chambers assembled with an airtight seal. A dispersion of microparticles is loaded into the upper chamber and let to diffuse for 24 h. (**B**) Direct evaluation of microparticle penetration (Dextran beads) across BC membranes. The upper surface of the BC membrane is reported as positive control (upper panel). The lower surface of the BC membrane upon incubation with Dextran beads (middle panel). The lower surface of BC membrane upon incubation with distilled water, reported as negative control (lower panel). (**C**) Bacterial colonization of pristine (upper left) and punctured BC membranes (lower left) and of the underlying agar substrate (right). A blue circle (lower right) indicates bacterial colonization underneath punctured BC membranes.

To demonstrate that the BC membranes under study establish an effective physical barrier against bacterial pathogens we initially evaluated their exclusion size in a permeation test in vitro. The results of this analysis, which exploited a custom developed two chamber cell (Fig. 3A), are reported in Fig. 3B. Microparticles of 2 µm in diameter were completely excluded from the lower chamber, indicating a cut-off size smaller than 2 µm. These data therefore demonstrate that the BC membrane structure is compatible with a barrier function against particles of this size or larger.

Next, the actual exclusion of bacteria from crossing the BC membranes was tested using inoculation with *S. aureus* (Fig. 3C). Specifically, bacteria were inoculated on the upper surface of BC membranes lying on agar plates. In this experimental configuration, the growth of bacteria on the underlying agar substrate demonstrates permeation across the test membrane.

In all tested samples, bacteria were able to grow and colonize the upper surface of BC membranes, indicating that cellulose is permeable to nutrients diffusing from the underlying plate (Fig. 3C). However, no signs of colonization were detected directly on the agar plate in the case of pristine BC barriers. On the other hand, when macroscopic holes were implemented on the membranes by puncturing them with a syringe, the BC layer became conducive to bacterial penetration and allowed colonization of the agar plate.

Altogether, these results demonstrate that BC membranes are permeable to small soluble molecules, but naturally constitute an effective physical barrier against the diffusion of microscopic structures, including bacteria.

### In vivo study set-up

To evaluate in vivo the protective effect of BC membranes towards bacterial colonization, we tested their barrier function upon the percutaneous implantation of L-VAD s.d.m.

Two groups of four adult female goats each entered the study. Each animal received 4 individual 6–10 cm long s.d.m. segments (see Materials and Methods). In particular, at each side of the animal one bare s.d.m. and one BC-coated s.d.m. were implanted percutaneously (Fig. 2B). Due to the additional thickness generated by the superposition of BC, percutaneous skin tunnels were distinctly larger for BC-coated s.d.m. than for their bare counterparts (Fig. 2B).

In general, wounds healed well in all animals. In case of wound secretion, implant sites were flushed with sterile 0.9% NaCl solution and wound dressings were changed. No animal had fever or signs of systemic infection during the observational period. The BC membranes gradually changed color (from opaque white to brown-yellow) along the duration of the study. Their distal part, which protruded outside of the skin, rapidly dried loosing flexibility and becoming brittle. The experiment was terminated at two subsequent time points, with the sacrifice and explantation at 6-weeks for the animals of the first group and at 12-weeks for those in the second group.

### In vivo barrier function of BC

After the subcutaneous tunnels were surgically prepared, an initial test was performed to evaluate the baseline contamination of the implant sites, and in particular the effect of their preparation, cleaning, and disinfection. In this frame, sterile microbiological swabs were employed do detect fungal or bacterial contamination of the tunnels and their respective exit sites. Therefore, 32 swabs samples were collected for microbiological analysis from both animal groups (16 from 6-week group and 16 from 12-week group).

None of the swabs collected prior to s.d.m. implantation showed signs of fungal colonization. On the other hand, despite thorough skin disinfection and use of sterile surgical equipment, 19 (19/32; 59.38%) specimens were positive to bacterial growth after culturing, with an almost equal distribution between the 6- (9/16) and 12-week (10/16) group.

This demonstrates an existing bacterial contamination in the majority of the surgical sites, which has to be expected as the applied disinfection procedure can reduce the resident flora but not to eradicate all microbes.

In total, 32 specimens were collected for microbiological analysis from both animal groups (6- and 12-week) including 16 BC-coated s.d.m. (8 at 6 weeks and 8 at 12 weeks) and 16 bare counterparts. Prior to sonication and subsequent plating, BC-coated s.d.m. were divided into their components (i.e. the BC and the s.d.m.) resulting in a total of 48 distinct specimens. Accordingly, for the analysis we considered the following target substrates: the control bare s.d.m. (B-s.d.m.; n = 16), the BC membranes (BC; n = 16), and the BC-deprived bare s.d.m. (BCB-s.d.m.; n = 16). Samples were considered colonized if bacterial growth was observed in agar plates inoculated with the sonication fluid after 24 h at 37 °C.

**Figure 4:**
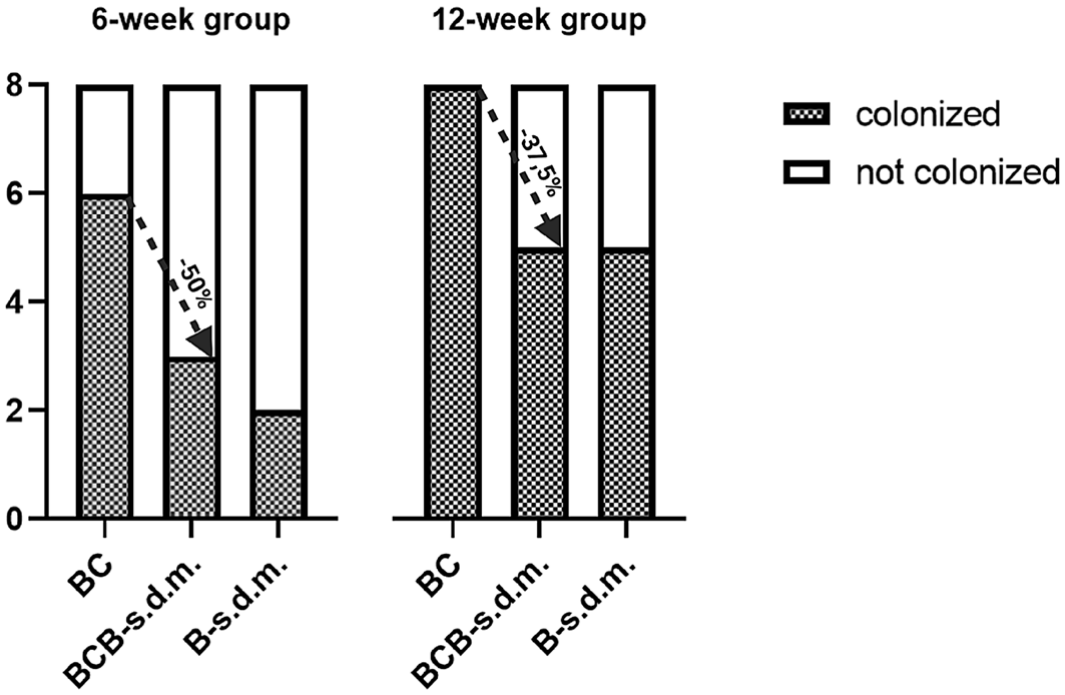
Microbiologic results after sonication and subsequent plating. Observed implant colonization rate (bacterial growth) in the 6- and 12-week-group. In both groups, bacterial colonization rate of BC was higher than for the underlying BCB-s.d.m. No corresponding BCB-s.d.m. colonization was observed in 50% and 37,5% of the cases in the 6- and 12-week group, respectively. The control group (B-s.d.m.) is reported as control.

As expected, due to the distinctly larger tunnel size the highest colonization rate occurred in the BC-group, where 75% (6/8) and 100% (8/8) of samples were colonized at 6 and 12 weeks, respectively (Fig. 4). The smaller tunnel size in the control group (B-s.d.m.) consistently reduced the colonization rate to 25% (2/8) and 37.5% (3/8) at 6 and 12 weeks, respectively. In all three target substrates the colonization rate increased between 6- and 12-weeks, with the higher colonization rates found in the 12-week group.

Despite colonization of the external BC cover, a significant fraction of the corresponding s.d.m. were free from bacteria. In fact, in 50% (3/6 at 6 weeks) and 37,5% (3/8 at 12 weeks) of the cases we registered no bacterial colonization of the corresponding BCB-s.d.m. (Fig. 4). Bacteria colonizing the external BC wrapping were therefore prevented from reaching the interface with the underlying s.d.m.

## Conclusions

Bacterial colonization of synthetic implant interfaces represents an outstanding biomedical challenge. The complex physicochemical material properties together with their intrinsic texture at the micro and nanoscale generate niches for bacterial growth, where the reach of the immune system is limited, and chronic infection can arise. Such complications often appear in conjunction with the intrinsic inflammatory reaction triggered by the interaction between the implant biomaterials and the surrounding tissues, leading to fibrotic tissue deposition. The formation of a fibrotic capsule, featuring poor vascularization and dense matrix, further contributes with the reduction of immune clearance, creating an ideal environment for bacterial growth.

BC has been recently proposed as viable protection against foreign body reaction, facilitating the fast regeneration of soft tissue in the surgical pocket of pacemakers^13^ or during the healing of skin wounds^17^. This natural hydrogel is well tolerated by the body for its mechanical and chemical properties, and has a typical nanofibrous composition, which naturally enforces a barrier to the penetration of inflammatory cells^13,17^.

The exclusion size of BC membranes is compatible with its function as a barrier against smaller cells (Fig. 3). While the intrinsic porosity allows for percolation, and thus diffusion of soluble molecules, it completely blocks the diffusion of microscale beads (Fig. 3B) and, more importantly, of bacteria (Fig. 3C). In vitro experiments clearly showed that bacteria cannot penetrate BC membranes. This effect is obtained by the typical pore diameter provided by the material which is well below the hindrance of bacteria. In fact, only when the integrity of the membranes is artificially compromised, by punching a microscopic hole in the BC layer, the underlying agar culture is colonized (Fig. 3C).

When such configuration is applied to L-VAD drivelines and implanted percutaneously in test animals, it reproduces its barrier function. Here, the external surface of the BC membranes is frequently colonized (Fig. 4). This is to be expected, in light of the site contamination prior to implantation and the relatively large diameter of the exit sites. The relevant result, which agrees with the in vitro permeation tests, is that in several cases the underlying surface of the s.d.m remains free from pathogens. We ascribe this effect to the exclusive effect of the BC porous structure, which restricts the contamination to its external surface.

It is indeed preferrable to limit bacterial contamination on the external surface of the BC membrane, rather than on other non-porous implant materials such as metals or silicones. Here, antimicrobial treatments are significantly more effective in eradicating bacterial colonization before a biofilm is produced^19^. This is again to be referred to the intrinsic porosity of the BC membranes, offering a percolating system, which allows the free movement of small soluble molecules (Fig. 3) but restricting the passage of larger biological moieties, such as human cells^13^ or bacteria (Fig. 3).

This observation, if confirmed, would open a way to avoid bacterial colonization of VAD drivelines and other exposed implant interfaces, limiting bacterial colonization to the protective BC membranes. BC can be generated in conformable layers that wrap and protect target implants, therefore forming an intervening level between the implant external interface and the surrounding tissue (Fig. 2). In light of the reduced fibrotic response against this material and the higher efficacy of antibiotic treatments against biofilm formation^19^, the presentation of bacteria and their clearance could be more efficient thus reducing or eliminating niches for bacterial growth.

This shall be regarded in the frame of an all-round implant protection, whereby the BC establishes an antifibrotic layer with the function of minimizing the foreign body reaction and promoting a prompt tissue regeneration^13^. In this scenario, potential contaminations may be managed better than in the case of a concomitant inflammatory process triggered by less compatible implant materials, such as the silicone polymers comprising the drivelines^19^.

## Limitations

For a proper interpretation of the results, some limitations of the study must be considered.

The differentiation of bacterial taxonomy among colonized specimens was not the aim of this study. However, it would be interesting to examine the correlations between bacterial species present at the implant site and those found on colonized implants. This correlation appears to be especially relevant considering that, despite skin disinfection with povidone-iodine solution, the majority of implant sites in our study were contaminated prior to implantation. Based on the observations reported in our work and other clinical studies which describe higher wound infection rates using povidone-iodine solution for surgical antisepsis, as compared to a chlorhexidine solution, a chlorhexidine-based skin disinfectant should be considered as antiseptic of choice in future studies^23^.

In addition, the cross section of percutaneous skin tunnels generated for the two conditions tested in this work (with and without BC coating) are different due to the increased diameter of the coated driveline mantles. Such increase may influence driveline infection rates^24^, therefore the study setup is not suitable for the evaluation of absolute colonization rates. The reduction of the exit site diameter shall represent a key factor to potentially reduce ensuing infection rates and facilitate the process of tissue regeneration. In this work, our focus was to demonstrate that BC established an impermeable layer which restricts contamination to the external surface of the implant, in contact with surrounding tissues.

Finally, the sites at which bacterial colonization originates in the coated driveline mantles are not known and the possibility that bacteria are present on the silicone surface before implantation cannot be excluded. This reduces the interpretation of the results to the conditions in which infections are detected on the external BC coating but not on the underlying driveline mantle.

## Supplementary Information

Supplementary Figure S1.

## Data Availability

The datasets generated during and/or analysed during the current study are not publicly available due to technical or time limitations but are available from the corresponding author on reasonable request.
