# Supplementary material for: On the function of biosynthesized cellulose as barrier against bacterial colonization of VAD drivelines
Source: Sci Rep. 2021 Sep 21;11:18776. doi: 10.1038/s41598-021-98220-4 (PMC8455583; doi:10.1038/s41598-021-98220-4)
Supplement: Supplementary file 1 — Supplementary Figure S1. [file 41598_2021_98220_MOESM1_ESM.docx]

**Supplementary Materials: On the function of biosynthesized cellulose as barrier against bacterial colonization of VAD drivelines -** Kaemmel et al.

**Manuscript ID:** f4e67203-5a0b-4df4-8895-7bad4aa17f0f

**
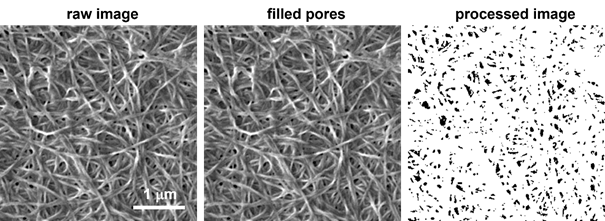
**

**Supplementary Figure 1** Image processing procedure for the calculation of pore diameter in BC membranes.
